# Association of socioeconomic status measures with physical activity and subsequent frailty in older adults

**DOI:** 10.1186/s12877-022-03108-1

**Published:** 2022-05-19

**Authors:** Mark Kheifets, Abigail Goshen, Uri Goldbourt, Guy Witberg, Alon Eisen, Ran Kornowski, Yariv Gerber

**Affiliations:** 1 Department of Cardiology, Rabin Medical Center, Petach Tikva; affiliated to the Faculty of Medicine, Tel Aviv University, Tel Aviv, Israel; 2 Department of Epidemiology and Preventive Medicine, School of Public Health, Faculty of Medicine, Tel Aviv University, Tel Aviv, Israel

**Keywords:** Exercise, Sports, Prevention, Frailty

## Abstract

**Background:**

Despite increased recognition, frailty remains a significant public health challenge.

**Objective:**

we aimed to assess the role of education and income, as well as neighborhood socioeconomic status, on physical activity and subsequent frailty in older adults.

**Methods:**

Using a population-based cohort of older adults, this study examined the relationship between socioeconomic status (SES) factors, physical activity and frailty. The study included 1,799 participants (mean [SD], 74.6 (6.2), 53.3% female) from the "National Health and Nutrition Survey of Older Adults Aged 65 and Over in Israel", conducted in 2005–2006. A follow-up interview was performed 12–14 years later in a subgroup of 601 subjects (mean [SD], age 84[4]; 56% women). Self-reported leisure-time physical activity (LTPA) was measured at both baseline and follow-up. SES measures were assessed at baseline. Frailty was measured at follow-up, using the Fried's Phenotype Model.

**Results:**

All SES measures were strongly and positively associated with LTPA (all *p* < 0.001). Eighty-two participants (14%) were classified as frail at follow-up. After age and sex adjustment and accounting for attrition bias using inverse probability weighting, baseline LTPA (OR = 2.77, 95% CI: 1.57–4.90, for inactivity; OR = 1.41, 95% CI: 0.75–2.68, for insufficient activity, compared with sufficient activity, P_trend_ < 0.001) was inversely associated with incident frailty. The association persisted after further adjustment for SES and comorbidity.

**Conclusion:**

Among older individuals, multiple SES measures were positively associated with LTPA, which was a strong predictor of lower subsequent frailty risk.

## Introduction

The term "frailty" is used to describe a range of conditions in older people, including general debility and cognitive impairment [1]. It is in fact a dynamic state, affecting an individual who experiences losses in one or more domains of human functioning (physical, psychological, cognitive and social) caused by the influence of a range of variables and which increases the risk of adverse outcomes [2, 3]. The relationships between demographic, socioeconomic status (SES), health-related, nutritional, and lifestyle factors and frailty are well established [4]. There is evidence to support the notion that neighborhood structural characteristics and social processes contribute to the development of frailty [5]. Moreover, there is an inverse association between frailty and both level of education and income [3, 4]. Given the increased recognition and awareness of frailty as a significant public health challenge, and its association with adverse health outcomes, prompt recognition, rapid diagnosis and both effective and efficient treatment are warranted [6, 7]. Although the critical time window for interventions has not yet been clearly established, as with various chronic diseases, primary prevention is the cornerstone of treatment in frail adult population. Raising awareness about the risk of the disabling cascade, providing the necessary knowledge to actively prevent, and improving access to care to favor optimal aging represent crucial steps to undertake [6]. The role of physical activity in the prevention and progression of frailty syndrome has been extensively researched, and is becoming increasingly well understood [7]. Even though existing evidence base is too inconsistent to recommend the optimal mode of a single physical activity or a single dietary regime for the prevention of frailty, there is emerging evidence for the synergistic benefits of combined physical activity and nutritional interventions for the older person living with frailty [7–9]. In regard to physical exercise, it improves the physical (cardiorespiratory function, muscle function, flexibility), cognitive and psychosocial state of frail individuals and consequently reduces the risk of adverse health outcomes, including mortality [10, 11]. Interestingly, physical inactivity serves as a stronger predictor for frailty in older adults, as compared to sedentary life style.

Meaning that physically active older adults have better physical function and frailty profiles than those considered physically inactive, even in the presence of high sedentary time [12, 13]. Although the effects of education, income and physical activity on frailty are well established, their influence on each other have yet to be studied. The aim of this present research was to evaluate the association of education and income, as well as neighborhood SES, on physical activity and subsequent frailty in older adults. Specifically, we utilized an extensive database of nearly 2,000 Israeli citizens aged ≥ 65 years.

## Methods

### Study design and setting

The study is a prospective cohort study investigating the role of sociodemographic, medical, and psychosocial variables in older adults. Details of the study methods have been previously reported [14]. Briefly, during 2005–2006, 1,799 older adults (mean [SD], age 74.6 (6.2) years, 53.3% female) participated in the First National Health and Nutrition Survey of Older Adults Aged 65 and over in Israel (‘Mabat Zahav’). The study was led by the Israel Center for Disease Control and the Nutrition Department of the Israel Ministry of Health. Data were obtained via a personal interview in the interviewee’s place of residence (own home or retirement home) using a structured questionnaire. Importantly, although this questionnaire was previously used in several studies, it hasn’t been validated for this specific population. The data collected in the survey (T1) included information regarding health and nutrition status, health behaviors (physical activity, alcohol consumption, medication use and use of nutrition supplements), knowledge and attitudes regarding nutrition and utilization of health services. During 2017–2019, a follow-up interview was conducted among 601 past participants (mean [SD], age 84.1[4.7] years, 55.9% female). The follow-up questionnaire (T2) duplicates most parts of the original (T1) interview. In addition, measurements pertaining to frailty status were performed according to Fried and colleagues’ Frailty Phenotype framework (FP) [15].

### Data collection

#### SES measures

Individual SES data were provided at baseline by self-report and included years of education, family status, current employment status (salaried, unsalaried or volunteer vs. none), and house-hold income as was measured by new Israeli shekels (NIS). Monthly household income was categorized, according to Israel minimum wage, as follows: low < 5,300 NIS (Monthly minimum wage for full-time employment; equivalent to 1,614$); intermediate 5,300–10,500 NIS; high > 10,500 NIS. Neighborhood SES was estimated according to home address, via an index developed and validated by the Israel Central Bureau of Statistics [16]. This index allows the classification of small geographic units into SES categories, based on socioeconomic measures (i.e., demographics, living standards, education, employment, social welfare benefits) from the 2008 National Census. Neighborhood SES scores were rated on a 20-point scale. A composite SES score (exposure variable) was calculated as follows. Education, income, and neighborhood SES were transformed into standardized z-scores, which were reversely coded such that negative values indicating better SES and positive values indicating worse SES. The composite SES score was computed by averaging the summed total. This particular score includes average data on education and income in the area of residence and not the detailed SES measures of the participants themselves.

#### Clinical variables

Self-rated health, a single question measure rated on a 4-point scale (4 -very good health) [17]. Mini mental state examination (MMSE) was adjusted for age and education [18]. General Health Questionnaire (GHQ) was used as a screening tool for common psychiatric conditions [19]. Hypertension was defined by one of the following: 1. Diagnosis of hypertension (given by a licensed physician) 2. Use of antihypertensive medication. Cardiovascular disease was defined by one of the following: 1. Diagnosis of ischemic heart disease, heart failure, peripheral vascular disease, cerebrovascular disease (given by a licensed physician) 2. Prior revascularization (either surgical or percutaneous). Function was assessed by the Katz Activities of Daily Living ADL scale based on ability to dress, shower/bathe, sit down and rise from a chair, eat, and go to the bathroom [20]. The maximum score is 15, with a score of 5 indicating "no functional limitations," 6–10 indicating "some functional limitations," and 11 or more indicating "severe functional limitations."

Each of the following diagnosis (given by a licensed physician) was defined as a comorbidity: diabetes, osteoporosis, hyperlipidemia, chronic renal failure, malignancy, glaucoma, cataract, Alzheimer's, Parkinson's, and chronic lung disease.

#### Primary exposure assessment

##### Physical activity

LTPA was self-reported during both baseline and follow-up interviews. The physical activity questionnaire used in the current study was based on a standard questionnaire used previously in an adult (aged 25–64) population study by the Israel Center for Disease Control performed together with the Food and Nutrition Services (for full questionnaire see Ministry of Health website – English version available [21]. In 2 sets of questions, participants were asked about their PA habits. One set referred to vigorous-intensity activity and another set addressed any type of moderate PA that lasted at least 10 min. Participants reported the frequency (times per week) and average time they devoted to each specific activity, as follows: walking outdoors or on a treadmill, jogging, swimming, bike riding or stationary cycling, light exercise (such as yoga, the Feldenkrais method, the Alexander technique, light gymnastics), body shaping, and strength training; an “other activity” option was also offered [22, 23]. Based on reported total weekly time of PA and intensity, participants were classified into 3 PA categories according to the official American College of Sports Medicine (ACSM) guideline [24]: sufficiently active, insufficiently active, or inactive. Individuals who performed moderate PA for at least 150 min per week or a vigorous-intensity activity for at least 75 min per week or a combination of the two were classified as sufficiently active; those who engaged in PA but in a lesser amount than these definitions were classified as insufficiently active; and those who reported no activity or activity less than once a week were classified as inactive.

#### Primary outcome assessment

##### Frailty

Frailty was assessed at T2 by the Fried's Phenotype Model [15]. Using this instrument, frailty was identified by the presence of three or more of the following components: 1. *Shrinking:* weight loss, unintentional, of more than 4.5 kg, or more than 5% of body weight, in the previous year; 2. *Weakness*: grip strength in the lowest 20% (adjusted for sex and body mass index); 3. *Poor endurance and energy*: as indicated by self-report of exhaustion; 4. *Slowness*: the slowest 20% of the participants in the sample, based on time of a 5-m walk (adjusted for sex and standing height); 5. *Low physical activity level*: a weighted score of kilocalories expended per week, based on the physical activity scale for the elderly (PASE) questionnaire [25]. The lowest quintile of physical activity was identified for each sex.

### Statistical analysis

Analyses were performed using IBM SPSS V.27 and R version 3.4.4 (R Development Core Team). Descriptive statistics of baseline characteristics of study participants by LPTA categories were compared by chi-squared test for categorical variables and analysis of variance for continuous variables. Logistic regression models were constructed in order to assess the role of the physical activity in long-term incidence of frailty. Logistic regression models were also used to assess the association between SES and incidence of frailty, before and after adjustment for LTPA. Missing values for covariates and individual components of the FP were imputed using multiple imputation methodology. Five datasets were created, with missing values replaced by imputed values based on models incorporating demographic, socioeconomic, psychosocial, and clinical variables. The results of these datasets were then combined using Rubin’s rules. Of the 1,799 participants in the initial survey, many were unable or unavailable to participate in the second interview. Because frailty could not be assessed among the latter group, selection bias is introduced. This bias was addressed through an adaptation of a marginal structural model, applying inverse probability weights [26]. The weights were calculated using logistic regression model to assess the probability of original participants to participate in T2. Each observation was then weighted by the reciprocal (i.e., the inverse) of the predicted probability of participating at T2.

## Results

Baseline Characteristics, categorized by LTPA at study entry, are shown in Table 1. Sufficiently active participants were younger, predominantly male, and mostly married. They had lower body mass index (BMI), smoked less, and suffered from less cardiovascular diseases. They had less comorbidities, less functional limitations, and rated their overall health higher, as compared to both inactive and insufficiently active participants.

**Table 1:** Baseline characteristics, categorized by LPTA at study entry

Variable	**LPTA categories**			*P* value
	Inactive (*n* = 734)	Insufficiently active (*n* = 506)	Sufficiently active (*n* = 559)	
Age, years, mean (SD)	75.1 (6.6)	74.8 (6.0)	73.9 (5.8)	0.002*
Female, n (%)	439 (59.8)	284 (56.1)	235 (42.0)	< 0.001
Employment, n (%)	176 (23.9)	106 (20.9)	113 (20.2)	0.097
Living alone n (%)	183 (24.9)	122 (24.1)	122 (21.8)	0.417
Married, n (%)	427 (58.5)	310 (61.6)	403 (73.1)	< 0.001
Self-rated health- good/ very good, n (%)	296 (40.3)	281 (55.5)	409 (73.2)	< 0.001
Cardiovascular disease, n (%)	296 (40.3)	171 (33.8)	180 (32.2)	0.002
Hypertension, n (%)	288 (40.1)	209 (41.6)	254 (45.7)	0.127
Comorbidities, n (%)				< 0.001
0	75 (10.2)	68 (13.4)	82 (14.7)	
1–3	538 (73.3)	366 (72.3)	435 (77.8)	
≥ 4	121 (16.5)	72 (14.2)	42 (7.5)	
BMI (kg/m^2^), mean (SD)	30.3 (5.4)	29.1 (4.5)	28.0 (4.0)	< 0.001**
Adjusted MMSE score, mean (SD)	30.5 (3.6)	30.8 (3.8)	30.8 (3.8)	0.213
No functional limitations*, n (%)	514 (70.0)	445 (88.0)	533 (95.3)	< 0.001
GHQ score, mean (SD)	6.8 (5.5)	6.7 (2.8)	5.6 (3.1)	0.034

Values are expressed as n (%) or mean ± SD.* According to Katz's ADL score ≤ 6

*Abbreviations: LPTA* leisure time physical activity, *BMI* body mass index, *MMSE* mini-mental state examination, *GHQ* general health questionnaire

^*^ANOVA post-hoc analyses (Bonferroni) significant < 0.05 difference between Inactive and Insufficiently active Vs. Sufficiently active LPTA categories

^**^ANOVA post-hoc analyses (Bonferroni) significant < 0.05 difference between all 3 LPTA categories

Baseline characteristics across baseline LTPA categories among T2 participants are shown in Table 2. Sufficiently active participants were predominantly male, mostly married, and were less likely to live alone. They had lower BMI, less comorbidities, and less functional limitations, as compared to both inactive and insufficiently active participants.

**Table 2:** Baseline characteristics, across baseline LTPA categories, among T2 participants

Baseline variable	**LTPA categories**			*P* value
	Inactive (*n* = 191)	Insufficiently active (*n* = 178)	Sufficiently active (*n* = 232)	
Age, years, mean (SD)	72.0 (4.8)	72.4 (4.7)	71.7 (4.5)	0.301
Female, n (%)	127 (66.5)	109 (61.2)	100 ((43.1	< 0.001
Living alone n (%)	40 (20.9)	41 (23.0)	31 (13.4)	0.027
Employment, n (%)	34 (18.0)	67 (37.6)	90 (38.8)	< 0.001
Married, n (%)	126 (66.0)	127 (71.3)	190 (81.9)	< 0.001
Self-rated health- good/ very good, n (%)	105 (55.0)	124 (69.7)	201 (86.6)	< 0.001
Cardiovascular disease, n (%)	50 (26.2)	40 (22.5)	62 (26.7)	0.516
Hypertension, n (%)	102 (53.4)	99 (55.6)	126 (54.3)	0.885
Comorbidities, n (%)				0.346
0	21 (11.0)	19 (10.7)	31 (13.4)	
1–3	150 (94.2)	147 (82.6)	188 (81.0)	
≥ 4	20 (10.5)	12 (6.7)	13 (5.6)	
BMI (kg/m^2^), mean (SD)	30.1 (4.8)	29.0 (4.0)	28.1 (3.9)	< 0.001*
Adjusted MMSE score, mean (SD)	31.0 (4.3)	30.8 (2.8)	31.2 (2.8)	0.543
No functional limitations, n (%)	164 (85.9)	173 (97.2)	229 (98.7)	< 0.001
GHQ score, mean (SD)	4.6 (3.9)	4.6 (3.5)	4.0 (3.4)	0.186

Values are expressed as n (%) or mean ± SD

*Abbreviations: LPTA* leisure time physical activity, *BMI* body mass index, *MMSE* mini-mental state examination

^*^ANOVA post-hoc analyses (froni) significant < 0.05 difference between Inactive Vs. Insufficiently active And Sufficiently active LPTA categories

SES measures, categorized by LTPA, at both baseline and follow-up are shown in Table 3. Sufficiently active participants were more educated, had a higher household income, and lived in neighborhoods with a higher SES score, as compared to both inactive and insufficiently active participants.

**Table 3:** SES measures, categorized by LTPA, at both baseline and follow-up

**Baseline LTPA Level (*n* = 1799)**				
SES measure	Inactive	Insufficiently active	Sufficiently active	*P* value
	(*n* = 734)	(*n* = 506)	(*n* = 559)	
Education, years, mean (SD)	8.7 (5.5)	11.1 (4.7)	12.5 (4.5)	< 0.001**
Household income category, n (%)				< 0.001
Low	381 (51.9)	201 (39.7)	189 (33.8)	
Intermediate	126 (17.1)	121 (23.9)	178 (31.8)	
High	223 (30.3)	182 (35.9)	190 (33.9)	
Neighborhood SES score, mean (SD)	9.4 (4.2)	11.1 (4.2)	11.5 (3.9)	< 0.001*
**Baseline LTPA Level among T2 participants (*n* = 601)**				
SES measure	Inactive (*n* = 191)	Insufficiently active (*n* = 178)	Sufficiently active (*n* = 232)	*P* value
Education, years, mean (SD)	8.4 (5.6)	12.0 (4.7)	12.4 (4.3)	< 0.001*
Household income category, n (%)				< 0.001
Low	94 (49.2)	61 (34.3)	56(24.1)	
Intermediate	31 (16.2)	45 (25.3)	81 (34.9)	
High	66 (34.6)	72 (40.4)	95 (40.9)	
Neighborhood SES score, mean (SD)	9.4 (4.4)	11.5 (4.5)	12.0 (4.1)	< 0.001*

Values are expressed as n (%) or mean ± SD

*Abbreviations: LTPA* leisure time physical activity, SES socioeconomic status

Household income categories: low < 5,300 NIS. Intermediate 5,300–10,500 NIS. High > 10,500 NIS

^*^ANOVA post-hoc analyses (Bonferroni) significant < 0.05 difference between Inactive Vs. Insufficiently active and Sufficiently active LTPA categories

^**^ANOVA post-hoc analyses (Bonferroni) significant < 0.05 difference between all 3 LTPA categories

**Fig. 1:**
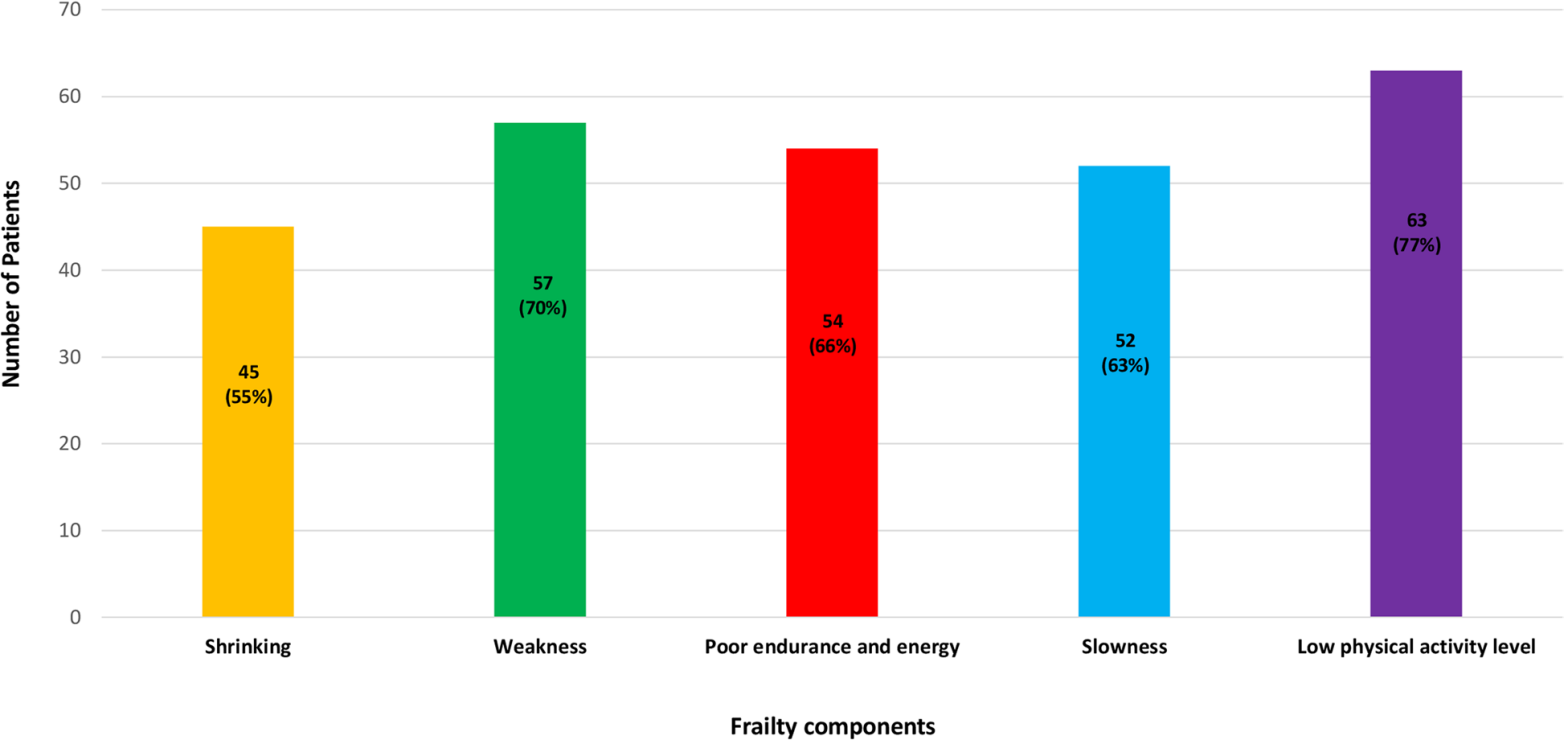
Frailty components among frail participants

Eighty-two participants (14%) were classified as frail at follow-up. Frailty components among frail participants are shown in Fig. 1.

Odds ratios (OR) of frailty development, according to baseline LPTA categories, are shown in Table 4. After adjustment for age and sex, decreasing LTPA levels were strongly associated with frailty incidence. Further adjustment for a composite SES score and comorbidity burden attenuated, but did not eliminate, the association. The distribution [mean z-score (SD); inversely coded] of the composite SES score by baseline LTPA categories was as follows: -0.32 (-0.31) for sufficiently active, -0.19 (0.57) for insufficiently active, and 0.34(0.69) for inactive (*P* < 0.001). Adjusted for age and sex, the OR (95% confidence interval) for frailty at follow up associated with lower SES was 2.25 (1.62–3.12). Further adjustment for PA attenuated the OR to 1.87 (1.01–3.46). Thus, approximately 20% of the SES-frailty association is attributable to PA.

**Table 4:** Odds ratios of frailty development, categorized by baseline LPTA, among study participants

***Adjustment***	**Physical activity categories**			**P for trend**
*Frailty phenotype*	Sufficiently active (*n* = 232)	Insufficiently active (*n* = 178)	Inactive (*n* = 191)	
*Model 1*	1	1.41(0.75–2.68)	2.77(1.57–4.90)	< 0.001
*Model 2*	1	1.32(0.70–2.53)	1.87(1.01–3.46)	0.04
*Model 3*	1	1.26(0.65–2.45)	1.71(0.90–2.24)	0.06

*Abbreviations: LPTA* leisure time physical activity, *SES* socioeconomic status

*Model 1:* age and sex. *Model 2:* further adjusted for SES composite score*. Model 3:* further adjusted for comorbidities

Comorbidities: heart attack, cardiac insufficiency, other heart disease, stroke, cataract, glaucoma, chronic renal failure, cancer, Alzheimer's disease, Parkinson's disease, asthma, other lung disease, diabetes, osteoporosis, dyslipidemia, hypertension

## Discussion

Population aging is poised to become one of the most significant social transformations of the twenty-first century, with implications for nearly all sectors of society [27]. While one 70-year-old person may enjoy good health that enables them to remain active and to live without much health care support or intervention, a peer of the same age may face multiple chronic morbidities that cause significant disability and require frequent medical interventions or various support resources. Level of income, educational attainment and physical activity may help to distinguish between the two. Approximately a quarter of individuals aged > 85 years are living with frailty and as such the identification of those who are frail is a public health priority [7].

In this large prospective registry of 1,799 older adults, 601 of which were interviewed for the second time after nearly 15 years, sufficient LTPA was associated with lower rates of frailty, as compared to both relative and absolute inactivity. Sufficiently active participants were younger, predominantly male, mostly married, had lower BMI, less comorbidities, lower rate of cardiovascular diseases, less functional limitations, were more educated, had a higher household income, and lived in neighborhoods with a higher SES score, as compared to both inactive and insufficiently active participants. Our study concluded that education and income, as well as area-based SES, serve as strong predictive factors for physical activity frequency, and subsequent development of frailty. Although various studies emphasized the powerful association between education, income and neighborhood SES in both vigorous [28] and LTPA [29], to our knowledge, this is the first study to describe the association between SES, physical activity and subsequent frailty in older individuals.

Our findings are consistent with earlier studies [2–4], which showed that physical activity is a key factor in both prevention and deceleration of the inevitable progression of an already established state of frailty. As was described by Van Oostrom et al. [3], being physically active decreases the risk of being frail on all four domains—physical, psychological, cognitive, and social. Furthermore, as was described by Woolford et al. [7], the degree to which one is physically active can directly contribute to the frailty syndrome in several ways. First, physical inactivity can lead to a myriad of diverse chronic health issues, including cardiovascular disease, cerebrovascular disease, type two diabetes, depression and dementia. Second, loss of muscle strength and progression to sarcopenia, may lead to imbalance, poor posture and eventual state of recurrent falls, with its potential adverse sequelae in the form of bone fracture, hospital admission and further decompensation. Combination of aerobic, resistance, balance, flexibility, and functional based exercise can help prevent the above. Therefore, it is crucial to follow a strict exercise prescription in order to avoid, and in some cases even reverse, frailty. As described by bray et al. [30], optimal frequency for multi-component training is 2–3 times per week. In addition, it is critical that individuals engage in exercise at an intensity that will elicit a fitness benefit by overloading the desired physiological system, causing it to adapt to meet the needs of the exercise demand. Duration of each exercise may vary, with optimal time of 30–60 min per session. It is important to remember that although any amount of physical activity, as compared to sedentary life style, lowers the risk of frailty, moderate to vigorous intensity physical activity has the greatest effect on frailty, with an emphasis on older adults with multiple comorbidities, such as ischemic heart disease, heart failure, diabetes, peripheral vascular disease, hypertension, chronic lung disease, etc. [31].

Concomitantly to physical activity, demographic and socioeconomic factors such as education and household income serve as overwhelmingly important risk factors for the prediction of frailty [3, 4]. As such, they should be taken under great consideration by educators, medical providers, and both local and government officials. Awareness for health and physical activity should be taught from the earliest age possible, and accessibility to fitness facilities in low socioeconomic neighborhoods is of paramount importance. Nutrition is yet another key factor in the development of frailty, and its quality is directly connected to SES and income. As was described by French et al. [32], lower income households purchase less healthful foods compared with higher income households. Food purchasing patterns may mediate income differences in dietary intake quality. Malnutrition is highly prevalent among older adults and associated with a general decline in physical and mental functioning, higher hospitalization rate and increased mortality [33]. As emphasized in several clinical guidelines [34], adequate caloric intake, and both protein and vitamin D supplementation, when indicated, are essential for the prevention and progression of frailty in older adults. As was so eloquently described by WOO et al. [35], neither nutrition nor frailty are topics that the majority of physicians and researchers are familiar with, but considering the continued increase in life expectancy on the global level, much needs to be done to raise awareness of the clinical importance of both. This approach represents true patient centered care in directing the goal of health promotion and clinical care towards maintenance of physical and cognitive function.

Lastly, frailty is associated with significantly higher health care costs, independent from pure age and comorbidities [36]. This holds true for both community dwelling older adults [37] and hospitalized patients suffering from frailty [38]. Since the number of frail individuals in old age will increase considerably in the next decades for reasons of demographic ageing, the phenomenon of frailty will certainly require more attention from the health care systems of industrialized countries. Hence, encouragement and active promotion of LTPA should be a top priority for all medical practitioners, with an emphasis on those working with underprivileged populations.

### Limitations

Although all data were collected prospectively, the SES-LTPA association was processed using a cross sectional analysis, which naturally limits the causal inferences of this study**.** Second, we did not have information regarding the participants physical activity during follow-up, nor did we assess frailty at baseline. We assumed that there were a very small number of frail participants at baseline, who attended the follow-up visit more than a decade later. Third, our data relied on self-reported questionnaires, without physical examination, laboratory workup or further imaging studies, and even though it was previously used in several studies, it hasn’t been validated for this specific population. Finally, only ~ 1/3 of the original participants were re-interviewed, leading to a relatively small sample size at follow-up, and ultimately resulting in attrition bias. Nevertheless, this current study presented a well-defined cohort of nationally representative older adults with repeated measurements of aging indicators, evaluation of frailty, rich dataset with multiple and multi-level SES and clinical measures, a detailed LTPA questionnaire, and an up-to-date statistical analysis to minimize the effect of attrition and missing data.

## Conclusions

This is a first of its kind study, which was able to demonstrated the important association of predictive factors such as education and income, as well as area-based SES, on physical activity frequency, and subsequent development of frailty. Since physical activity is an effective and generally inexpensive form of both prevention and treatment of frailty, it should be a central emphasize of both policy makers and health care providers, especially in lower income areas.

## Data Availability

The datasets generated and analyzed during the current study are available in the "Mabat Zahav" repository (www.health.gov.il).

## Abbreviations

SES: Socioeconomic status
LTPA: Leisure-time physical activity
FP: Frailty Phenotype framework
MMSE: Mini mental state examination
ACSM: American College of Sports Medicine
BMI: Body mass index
PASE: Physical activity scale for the elderly
GHQ: General health questionnaire
OR: Odds ratio
